# Cumulative stress in research animals: Telomere attrition as a biomarker in a welfare context?

**DOI:** 10.1002/bies.201500127

**Published:** 2015-12-08

**Authors:** Melissa Bateson

**Affiliations:** ^1^ Centre for Behaviour and Evolution/Institute of NeuroscienceNewcastle UniversityNewcastle upon TyneUK

**Keywords:** animal welfare, biological age, biomarker, cumulative experience, cumulative severity, stress, telomere dynamics

## Abstract

Progress in improving animal welfare is currently limited by the lack of objective methods for assessing lifetime experience. I propose that telomere attrition, a cellular biomarker of biological age, provides a molecular measure of cumulative experience that could be used to assess the welfare impact of husbandry regimes and/or experimental procedures on non‐human animals. I review evidence from humans that telomere attrition is accelerated by negative experiences in a cumulative and dose‐dependent manner, but that this attrition can be mitigated or even reversed by positive life‐style interventions. Evidence from non‐human animals suggests that despite some specific differences in telomere biology, stress‐induced telomere attrition is a robust phenomenon, occurring in a range of species including mice and chickens. I conclude that telomere attrition apparently integrates positive and negative experience in an accessible common currency that translates readily to novel species – the Holy Grail of a cumulative welfare indicator.

**Abbreviations**
bp: base pairs
TA: telomere attrition (i.e. loss in TL between two measurements, measured in base pairs of DNA)
TL: telomere length (measured in base pairs of DNA)

## Introduction

Biologists working with live vertebrate animals are ethically obliged to optimise the welfare of their subjects, refining husbandry and experimental procedures to minimise the potential for pain, suffering, distress or lasting harm. In line with this principle, current European legislation governing the use of animals in research places an emphasis on the animal's lifetime experience, and requires both prospective and retrospective assessment of the cumulative effects of the research on welfare 1. But what exactly is meant by lifetime experience, and how should we measure it?

Animal welfare is assumed to be influenced by the cumulative effects of the positive and negative events experienced by an individual 2. The terms ‘cumulative severity’ and ‘cumulative experience’ are used to refer to the sum of all positive and negative impacts on the health and welfare of an animal over its lifetime 3. Negative impacts include not only the direct negative impacts of experimental procedures such as pain, fear or hunger, but also the negative impacts entailed in transport, handling and housing of experimental animals, and the negative impacts arising from complications such as infections or physical injuries. Positive impacts include refinements to breeding, husbandry or procedures that either directly improve the health and welfare of experimental animals or mitigate the negative impacts of experimental procedures. Cumulative experience has therefore been defined as: ‘the sum of all the events and effects, including their quantity, intensity, recovery between and memory thereof, that impact adversely, positively, and by way of amelioration on the welfare of an animal over its lifetime’ 3.

While the definition of cumulative experience appears relatively clear, the problem lies in how it should be assessed. Veterinarians and scientists faced with assessing cumulative experience currently resort to crude objective measures such as body weight, which lack the required levels of selectivity and sensitivity for detecting subtle changes in welfare, and to clinical impression, which is subjective and open to disagreement. More sophisticated schemes have been proposed, but these rely on untested assumptions about the magnitude of different impacts and how these add up 4. The crux of the problem is that we lack an objectively measurable common currency that is sensitive to the various positive and negative events that an animal experiences.

Here I argue that findings from the biology of cellular ageing suggest that telomere attrition (TA) is a candidate measure worthy of serious consideration. In 1956, Hans Selye wrote, ‘Every stress leaves an indelible scar, and the organism pays for its survival after a stressful situation by becoming a little older’ 5. Selye's observation that there are links between stress and ageing was prescient, and the intervening 60 years of research have borne out his idea that unpleasant stressors of various kinds accelerate ageing and increase mortality 6, 7, 8, 9, 10. These findings have led directly to the concept of biological age as distinct from chronological age 11. My thesis therefore is that the biological age of an animal could be used to determine its cumulative experience: animals that are biologically old for their chronological age have had relatively unpleasant lives compared with animals that are young for their chronological age. In the remainder of this essay, I explore the evidence that the cellular damage associated with ageing might yield an objective measure of cumulative experience that could be useful in an animal welfare context. Specifically, I focus on the evidence that telomere length (TL) is a biomarker linking stress to premature ageing, and argue that TA is a plausible candidate for the ‘indelible scar’ referred to by Selye.

In Section 2, I clarify the criteria for a good measure of cumulative experience in animals. In Section 3, I describe telomere biology and present evidence that TL should be sensitive to negative experiences associated with poor welfare. In Section 4, I review how telomeres are measured, and show that the methods meet the criteria established in Section 2. In Section 5, I review the epidemiological evidence demonstrating the association between TL and the valence (pleasantness/unpleasantness) of experience in humans. In Section 6, I review the comparative biology of telomere dynamics in non‐human vertebrates and conclude that the findings from humans are plausibly applicable across many non‐human species for which we have specific welfare concerns. In Section 7, I summarise the evidence suggesting that TA in non‐human vertebrates is sensitive to negative experiences likely to induce poor welfare. In Section 8, I describe a simple mathematical model that explicitly summarises how TA could integrate positive and negative experience in a common currency. I conclude in Section 9 and highlight the need for further experiments to test my proposal.

## Criteria for a good measure of cumulative experience

In assessing animal welfare, it is easy to fall prey to anthropomorphism and impose subjective judgements about whether a given experience is positive or negative 12. Not surprisingly opinions differ; a neurobiologist might argue that a chronic implant on a monkey causes minimal ongoing suffering, but an animal welfare campaigner will take a different view. Thus, a good measure of cumulative experience must be objective, and show intra‐ and inter‐observer reliability. To have any hope of actually being used in applied settings, a measure needs to be practical to acquire. Furthermore, it should be possible to measure in vivo, relatively non‐invasively, since a measure that can only be acquired post mortem, or that imposes significant additional stress, has limited use for monitoring the welfare of animals in long‐term experiments.

Perhaps the central criterion for a comparative measure of cumulative experience is that it should be based on a biomarker common to all vertebrate species that is likely to respond in a similar way across species to positive and negative experiences (behavioural measures of poor welfare are particularly problematic in this regard because they are often highly species‐specific). In order to provide the type of integrative accounting required, a measure needs to vary on a continuous scale, and respond in opposite directions to positive and negative experiences. The measure should be selective to the valence of experience and not sensitive to orthogonal attributes of experiences, such as, for example, level of arousal (a problem with stress hormones 13). Ideally a measure should be dose dependent, and hence sensitive to the duration and magnitude of experiences in addition to their valence. It must also be cumulative, and provide integration over time of positive and negative experiences, as opposed to merely a snap‐shot of current state.

A good measure of cumulative experience needs to be validated. However, the validation of any welfare measure is problematic because the process is prone to circularity: proving that a novel measure is sensitive to a positive or negative experience depends on knowing the valence of that experience, and if no good measures of experience currently exist, this is not straightforward. One solution is to establish the validation in humans. Humans are unique in being able to report the subjective emotional experience associated with an event and being able to describe their longer‐term moods 13. Moods are sensitive to the positive and negative experiences of an individual, and hence are likely to be a good reflection of cumulative experience 2, 14. Thus, we can use measures of human mood to validate objective measures of cumulative experience that are available in both humans and non‐human animals. Note that this proposal is not anthropomorphic. I am not assuming that animals necessarily experience subjective feelings like those of humans. Moreover, I am not assuming that an experience that is negative for a human will necessarily also be negative for an animal. I am merely arguing that a good comparative measure of cumulative experience fails at the first hurdle if it is not strongly correlated with mood in humans.

To summarise, I propose that a good biomarker of cumulative experience should be: objectively measureable in any species, selectively sensitive to the cumulative effects of positive and negative experiences in a dose‐dependent manner and correlated with mood in humans.

## Telomere dynamics: A primer

The brief review of telomere biology that follows focuses on what is known about telomeres in humans. My reasons for this are twofold: first, we know most about telomeres in humans, and second, the validation of TA as a measure of cumulative experience will be performed in humans, as described above.

Telomeres are DNA‐protein complexes at the ends of the chromosomes, and telomeric DNA comprises repeats of the TTAGGG nucleotide sextet. The function of telomeres is to protect the coding regions of DNA from damage during cell division and DNA repair. Due to the ‘end pair replication problem’, telomeres shorten with every cell division. Although telomeres can be repaired by the enzyme telomerase, this enzyme varies in abundance between species, tissues and over the course of development 15. In post‐natal humans, telomerase is repressed in somatic tissues and its levels are insufficient to offset TA. Therefore, in proliferating somatic tissues, telomeres shorten with age from birth onwards. Thus, TL provides a biological clock that effectively counts cell divisions.

Human telomeres start with an average length of 8–20 kb at birth. However, the rate at which the telomere clock ticks is not constant across the lifespan, and varies in different tissues. For example, leukocyte TA is very high in early life, corresponding to the period of most rapid cellular proliferation, and slows to a relatively constant rate in adulthood of approximately 30 bp per year on average 16, 17, 18. The pattern in muscle tissue is rather different, with a lower rate of attrition throughout the post‐natal lifespan 19. Although the rates of attrition in different tissues vary, there is typically a correlation between the average TLs in different tissues within an individual 20. When the telomeres of a cell become too short, it enters a senescent state and dies. Loss of cells via this route impacts the function of tissues and ultimately the organism, suggesting that TA is both a biomarker of cellular age but also potentially causal in the ageing process.

Central to the argument developed in this essay, the rate of TA in a given tissue at a given age is not fixed, but can be altered via a range of different cellular mechanisms 21. Inflammation causes TA in blood by increasing the rate of leukocyte turnover and hence increasing the rate of replicative senescence. Oxidative stress causes TA because free radicals directly damage the vulnerable G triplets of the telomeric sequence 22. Finally, cortisol causes TA by reducing telomerase activity and hence slowing telomere repair 23. Cortisol also increases free radical production and interferes with antioxidant defences, thus increasing oxidative stress within the cell 21.

Since rises in oxidative stress, inflammation and/or cortisol are associated with the types of negative life events assumed to contribute to poor welfare (Table 1), I propose that TA could provide a molecular read‐out of cumulative negative experience. In addition to the evidence summarised in Table 1, there is also evidence to suggest that active stress reduction is associated with opposite trends in the same physiological processes known to increase TA. For example, the practice of mindfulness‐based stress reduction in humans produces reduction in measures of both inflammation and cortisol 24.

**Table 1:** Examples of evidence from humans that the classes of stressors assumed to contribute to poor animal welfare are associated with physiological changes known to accelerate TA in vitro

Class of negative experience	Specific example	Physiological changes linked to TA	References
Physical injury	Acute spinal cord injury	Inflammation ↑	62
Infection	Bacterial pathogens	Inflammation ↑	63
Exposure to environmental toxins	Tobacco smoke	Inflammation and OS ↑	64
		Cortisol ↑	65
Pain	Fibromyalgia	Inflammation ↑	66
	Assorted types of chronic pain	Cortisol ↑	67
Disturbed sleep	Shift work	Cortisol ↑	68
Psychosocial stressors	Serious life events (death of a relative, divorce, etc.)	Cortisol ↑	69
Mood disorders	Anxiety disorders	Cortisol ↑	70
		Inflammation ↑	71
	Major depression	Inflammation and OS ↑	72
		Cortisol ↑	73

**Figure 1:**
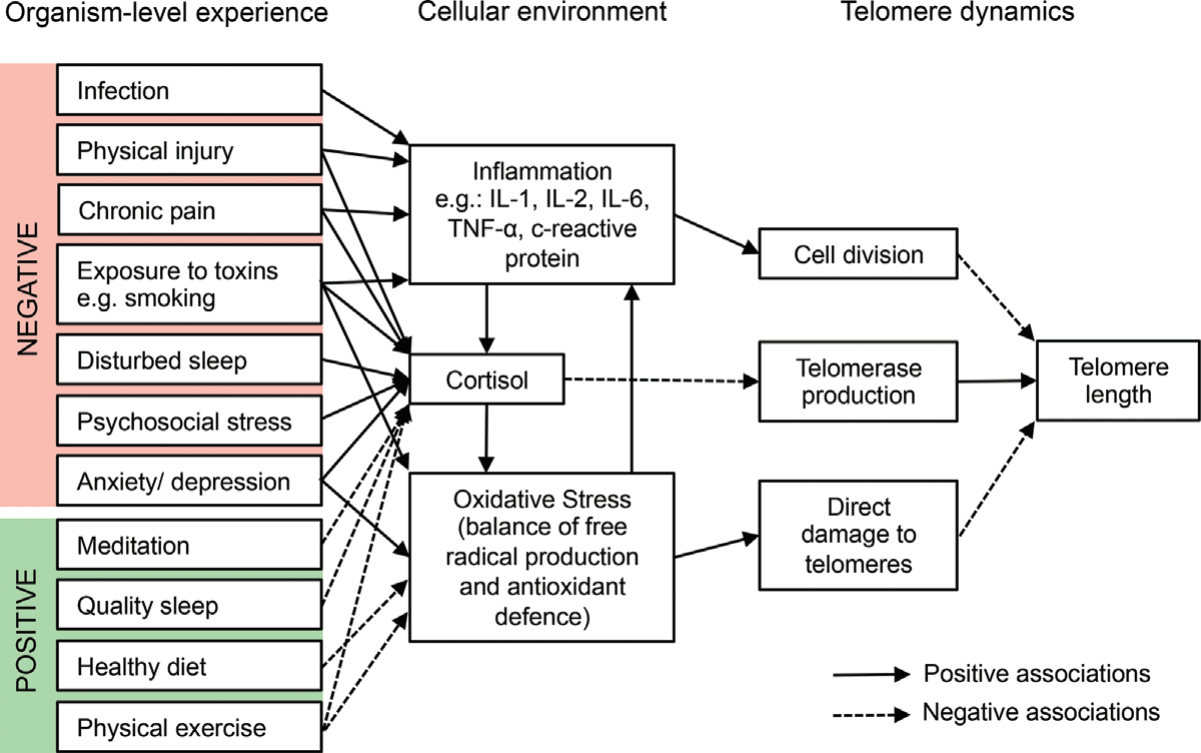
Summary of the some of the known relationships between organism‐level experiences, the cellular environment and telomere dynamics in humans. In general, it appears that negative experiences shorten telomeres, whereas positive experiences can retard, prevent or possibly reverse telomere attrition. References for the associations shown in this figure are given in Table 1 and Section 3 of the text.

In summary, human telomeres decline in length following birth and their rate of attrition should be sensitive to a range of organismal stressors that induce increases in inflammation, oxidative stress or cortisol (Fig. 1). Thus, TA is potentially a biomarker sensitive to life events assumed to contribute to mood.

## Measurement of telomeres

Since TA occurs fastest in rapidly dividing cells, samples for studies of stress‐induced TA need to come from proliferating tissues. In humans, the majority of studies of TL have been done on blood using either leukocytes (all white blood cells, predominantly neutrophils) or peripheral blood mononuclear cells (a subset of leukocytes, predominantly lymphocytes). Buccal cells have also been used in some studies where minimally invasive sampling is required 25, 26. All of these tissues can be collected in vivo meaning that longitudinal studies (in which a single individual is sampled repeatedly) are possible.

Several methods are available for measuring TL, the four most common being: quantitative real‐time PCR (QPCR) to measure the ratio of the abundance of the telomeric sequence to that of a single‐copy gene (which needs to be established for each species); analysis of terminal restriction fragments (TRF); single TL analysis (STELA) and fluorescence in situ hybridisation‐based (FISH) methods. All are capable of providing an estimate of TL in bp (QPCR provides a relative measure that can be converted to bp via the use of standards of known length). These methods are fully described and their pros and cons discussed elsewhere 27, 28. The optimal method depends on the nature of the scientific question, the species, the number of samples to be analysed and the size and type of the samples available 27, 28. QPCR is currently the only high‐throughput method for measuring TL and is commonly used in epidemiological studies. It requires less DNA than TRF and is less sensitive to DNA degradation. However, QPCR has the disadvantage of being less precise and providing only a single average TL estimate for a sample compared with the distribution of TLs yielded by the other methods 27. Although different methods and different laboratories produce different absolute estimates of TL, reassuringly, ranking of TL in the same set of samples is similar for different methods and laboratories 29.

There is considerable inter‐individual variance in TL in a given tissue in humans of the same age. This variance is present at birth (a range of 8.6–13.3 kb in 30), and heritability of human TL has been calculated at 78% 17, suggesting a strong genetic influence. Given this variability, attempts to link TL to lifetime experience ideally need to control for differences in birth TL. The best approach is to sample the same individual longitudinally. It has been estimated that due to inter‐individual variation in TL, longitudinal studies require five times fewer subjects than cross‐sectional studies to detect a given effect 31. An alternative (but more invasive) approach is to use TL in minimally proliferating tissues (such as skeletal muscle) as a proxy for birth TL 32.

In assessing the evidence for changes in TL, three caveats need considering. First, in cross‐sectional studies, the effects of within‐individual attrition with increasing age can be masked by the selective mortality of individuals with short telomeres. Second, apparent changes in TL in longitudinal studies, particularly when the interval between baseline and follow up samples is short, can be caused by measurement error 33, and corrections may be necessary to control for regression to the mean 34. Finally, even if changes in TL are real, the biological explanation needs to be considered. Since different cell types can have different TLs, it is possible for a change in the relative proportions of different cell types contributing to a sample to generate apparent change in TL 35. This problem can be addressed by using a more homogeneous cell population (e.g. erythrocytes in non‐mammals), or by demonstrating that proportions of different cell types are unchanged 36.

In summary, TL can be measured objectively with reasonable reliability using samples from tissues that can be obtained relatively non‐invasively in vivo. High inter‐individual variability in TL means that longitudinal studies, in which a single individual is sampled at least twice, are likely to be necessary to detect subtle effects of individual experience on TA.

## Telomere length in humans is sensitive to positive and negative experiences

In humans, it has been argued that TL could be used as a ‘psychobiomarker’ of cumulative lifetime exposure to stress 37. This view is supported by mounting evidence from epidemiological studies showing that people that have had various types of negative experiences have shorter telomeres than age‐matched controls, and that the effects might be dose dependent 38, 39. For example, anxiety 40, depression 41, neuroticism 42, pessimism 43, childhood exposure to violence 44, family disruption 25, chronic pain 45, 46 are all associated with shorter telomeres. In the first study of this type, Epel and coworkers 47 showed that TL in female caregivers was negatively correlated both with the number of years spent as a caregiver and the subjective perceived level of stress. The TLs in the women with the highest perceived levels of stress were equivalent on average to over a decade of additional ageing.

There is growing suggestion from longitudinal studies, comparing TL before and after stressors are experienced, that the above correlations are due to negative experiences actually causing TA 44, 48, 49. For example, in a prospective longitudinal study of 263 healthy middle‐aged women followed over 1 year, each additional major life stressor (events such as divorce, loss of household, death of a relative and unemployment) experienced during that period reduced TL by an additional 35 bp 49.

A good measure of cumulative experience should be sensitive to positive experiences as well as to negative experiences, since both ought to have an impact on resultant mood. There is recent evidence that a healthy lifestyle can mitigate the detrimental effects of stressful life events on TL 50. In the longitudinal study described above 49, TA was predicted by an interaction between the number of life stressors experienced and the healthiness of a woman's lifestyle, assessed via diet, sleep and physical exercise, whereby women with the healthiest lifestyles were immune to the telomere shortening effects of major life stressors. These data support the idea that the ageing effects of stressors could be mitigated by life‐style interventions.

Telomerase can repair telomeres, and there is some evidence that positive life‐style interventions, such as the practice of mindfulness meditation, are associated with increased telomerase levels 51. Therefore, it is theoretically possible that individual telomeres could lengthen. Although several longitudinal studies of telomeres report a percentage of subjects showing apparent telomere lengthening, many of these cases can be more parsimoniously explained as measurement error 33. However, where systematic telomere lengthening occurs within a study, measurement error is unlikely to account for this. A study comparing patients with current anxiety disorders, previous patients with remitted anxiety disorders and healthy controls found shorter telomeres in the current patients but no difference in TL between current patients and remitted former patients 40. Furthermore, they found that the time since remission was positively associated with TL in the remitted group. One explanation for this pattern is that TL gradually lengthened following remittance from anxiety.

In summary, there is abundant evidence that psychosocial stress and negative mood is associated with increased TA in humans. There is also growing evidence that positive life‐style interventions can mitigate the negative effects of stress on TA. It is still unclear whether TL can be increased via positive interventions, but this is theoretically possible and there is some evidence compatible with telomere lengthening. Since in humans both chronological age and effects of stress can be measured in bp of telomere, it is possible to express the impact of various life stressors in terms of the number of years of ageing they equate to. Therefore, TL is a plausible common currency with which to assess cumulative experience in humans.

## Telomere biology in non‐human species

Thus far I have focussed on telomere dynamics in humans. However, the translation of these findings to the assessment of cumulative experience in non‐human animals depends on whether similar telomere biology exists in other species.

Telomeres occur in all eukaryotic cells, and the telomeric sequence, TTAGGG, is evolutionarily conserved in vertebrates. However, there is variation between species in potentially important aspects of telomere biology including mean TL, and whether telomerase production is repressed in somatic tissues following birth. The human pattern, consisting of relatively short telomeres and repressed telomerase in somatic tissues following birth, is probably ancestral within the mammals, and has been retained in most large, long‐lived mammals 15, 52. However, this pattern has been lost in some smaller‐bodied mammals including laboratory rats and mice, which have long telomeres and telomerase‐based telomere maintenance in somatic tissues throughout life 52. Fish, amphibians and reptiles all have telomerase‐based telomere maintenance in somatic tissues throughout life 53. Birds appear to have independently evolved a more human‐like pattern with telomerase repression in somatic cells following hatching 54. Table 2 presents comparative telomere biology in selected common laboratory, farm and companion animal species that currently attract the majority of welfare‐related research (henceforth, species of welfare concern).

**Table 2:** Comparative telomere biology in selected farm, laboratory and companion animals

Taxonomic group	Species^a^	Nucleated erythrocytes	TL (kb)^b^	Telomerase^c^	Age‐related TA^d^	Key references
Primates	Human (*Homo sapiens*)	No	8–20	No	Yes (long)	See main text
	Macaque monkeys (*Macaca sp*.)	No	15–18	No	Yes (cross)	32, 74
Rodents	Mouse (*Mus musculus*)	No	25–200 (laboratory)	Yes		75, 76
			12–25 (wild derived)			
Lagomorphs	Rabbit (*Oryctolagus cuniculus*)	No	20–50	No		77
Carnivores	Domestic dog (*Canis lupus*)	No	10–23	No	Yes (cross)	78, 79, 80
	Domestic cat (*Felis catus*)	No	5–26	No	Yes (cross)	81
Cetartiodactyles	Cow (*Bos taurus*)	No	18	No	Yes (cross)	82, 83
	Sheep (*Ovis aries*)	No	9–23	No	Yes (cross)	84, 85
	Pig (*Sus scrofa domestica*)	No	10–30	Unclear	Yes (cross)	86, 87
Perissodactyles	Horse (*Equus caballus*)	No	14	No	Yes (cross)	88, 89
	Donkey (*Equus asinus*)	No	7–21	No	Yes (cross)	90
Birds	Parrot (*Psittacus erithacus*)	Yes			Yes (cross)	91
	Chicken (*Gallus gallus*)	Yes	0.5–4000	No	Yes (cross)	54, 92, 93
	Zebra finch (*Taeniopygia guttata*)	Yes	8–30	No	Yes (long)	94, 95, 96, 97
	European starling (*Sturnus vulgaris*)	Yes			Yes (long)	59, 60, 98
Teleost fish	Salmon/trout (*Oncorhynchus/Salmo*)	Yes	20	Yes	No (long)^e^	99, 100, 101, 102
	Zebra fish (*Danio rerio*)	Yes	2–10	Yes		56

Blank cells indicate that I have not been able to find any relevant data.

a I have selected a few species judged to be of particular welfare concern for this table. Data on many more species including some common zoo animals are available in the following reviews: 15, 52, 53, 103.

b TL estimates come from a variety of methods and the ranges reported span values given in the papers reviewed.

c This column records whether telomerase is expressed in adult somatic cells. For mammals (excluding cat and dog), data are taken from 15. For other species, data are taken from sources cited in the final column.

d This column records whether age‐related TA has been documented from blood/tissue samples in vivo: ‘cross’ indicates a cross‐sectional study and ‘long’ a longitudinal study (the better method). Sources are cited in the final column.

e This result is for wild‐type fish; age‐related TA was found in transgenic salmon with an artificially increased growth rate 99.

Telomere length and distribution (specifically whether interstitial repeats of the telomeric sequence are present within chromosomes) both affect the optimal method for measuring telomeres 28. Very long telomeres are more prone to shearing (e.g. during DNA extraction) and whilst this will have less effect on estimates of TL derived from QPCR, it will bias estimates from TRF. Interstitial telomere repeats will contribute to the mean TL estimated using QPCR, but not via some TRF techniques. However, this latter problem is not important if QPCR is being used for longitudinal measurements, because TA occurs only at the ends of chromosomes 28. Chickens have both very long telomeres and interstitial repeats, meaning that the most common method for measuring TL in this species has been FISH; no published chicken studies have used QPCR. In discussing the measurement of TL, another point worth mentioning is that birds, fish, amphibians and reptiles differ from mammals in having nucleated erythrocytes. Since erythrocytes comprise 99% of blood cells, much less blood is required for TL measurement in non‐mammalian species, facilitating longitudinal sampling in small animals. Erythrocytes also constitute a more homogenous population of cells than leukocytes, meaning that changes in TL measured in erythrocytes are simpler to interpret.

Telomere length and, the extent to which telomerase is repressed in somatic tissues, are both likely to affect the in vivo pattern of telomere dynamics observed within a species. Age‐dependent TA will be greater in species that repress telomerase. Stress‐induced TA could be harder to detect in species with very long telomeres, because a given amount of loss would represent a smaller proportion of the total telomeric DNA present. Stress‐induced TA could also be harder to detect in species that retain telomerase expression, because of the greater potential for telomere repair. For these reasons, the laboratory mouse was, until recently, thought to be a poor model for studying in vivo telomere dynamics 55. It is also possible that fish will not show stress‐induced TA because, despite having relatively short telomeres, they retain telomerase expression as adults 56.

In summary, there is variation between species in TL and the extent to which telomerase is repressed in adult somatic tissues. This variation affects the optimal method for measuring TL, but more importantly it could also affect telomere dynamics in vivo. Reassuringly for my proposal, the majority of species of welfare concern share the human‐like pattern of relatively short telomeres coupled with telomerase repression, leading us to predict age‐ and stress‐induced TA in these species. However, in chickens, mice and fish, there are substantial differences in telomere biology compared with humans that might lead us to expect a reduced impact of stress‐induced TA in these species.

## Evidence for stress‐induced telomere attrition in non‐human vertebrates

Recent literature supports the idea that various types of stress likely to be associated with poor welfare are associated with shorter telomeres or increased TA in a range of non‐human species (Table 3). Notably, these species include the mouse and the chicken, two species whose telomere biology is substantially different from that of humans (Table 2). Thus, despite concerns to the contrary, stress‐induced TA can occur even in species such as the mouse, which has adult telomerase expression, presumably via the direct action of corticosterone on telomerase. It also seems that long telomeres (in laboratory mice and chickens) do not prevent the measurement of stress‐induced TA. To date, the only evidence for stress‐induced TA in fish comes from genetically modified salmon 99; it remains to be shown whether stress‐induced TA occurs in wild‐type fish.

**Table 3:** Examples of studies investigating effects of various types of stress on TA in selected laboratory, farm and companion animals

Species	Age^a^ and sex	Type of study^b^	Tissue	Method	Number of subjects	Stress manipulation	Control	Effect of stress on TL	Refs.
Wild house mouse (*Mus musculus*)	Juvenile females (1–6 months)	Expt	Leukocyte	QPCR	8 trt	Rapid reproduction for 5 m (offspring removed)	Pair of females	↓	55
		Long: 5 months			20 con				
Wild house mouse (*Mus musculus*)	Juvenile females (1–6 months)	Expt	Leukocyte	QPCR	9 trt	Crowding for 5 m (offspring not removed)	Pair of females	↓	55
		Long: 5 months			20 con				
Wild house mouse (*Mus musculus*)	Juvenile males (1–6 months)	Expt	Leukocyte	QPCR	10 trt	Crowding for 5 m (offspring not removed)	Male–female pair (offspring removed)	↓	55
		Long: 5 months			8 con				
Wild house mouse (*Mus musculus*)	Adults (3–12 months)	Expt	Leukocyte	QPCR	24 trt	5× repeated infection with *Salmonella enterica*	5× oral saline	↓ In males	61
		Long: 8 months			24 con				
Laboratory mouse C57BL/6J (*Mus musculus*)	Adults (12 months)	Expt	Leucocyte (blood and saliva)	QPCR	16 trt	4× repeated cycle of tail suspension, forced swim, foot shock, restraint and sleep deprivation	No stress	↓	36
		Long: 4 weeks			16 con				
Laboratory mouse C57BL/6J (*Mus musculus*)	Adult females (12 months)	Expt	Leucocytes (blood and saliva)	QPCR	8 trt	Daily injection of 30 mg/kg corticosterone	Daily injection of vehicle (oil)	↓	36
		Long: 4 weeks			8 con				
Sudanian grass rat (*Arvicanthis ansorgei*)	Juvenile males (2–3 months)	Expt	Hepatic cells	QPCR	12 trt	Chronic ‘jet lag’ (bi‐weekly peturbations of LD cycle) for 30 days	12:12 hours LD cycle for 30 d	↓	104
		Cross			12 con				
African grey parrot (*Psittacus erithacus*)	Adults (0.75–45 years)	Obs	Erythrocyte	QPCR	26 trt	Socially isolated	Pair‐housed	↓	91
		Cross			19 con				
Domestic broiler chicken Ross 308 (*Gallus gallus domesticus*)	Chicks (7 days)	Expt	Lymphocyte	FISH	8 trt	High stocking density (0.06 m^2^/bird) for 28 days	Low stocking density (0.1 m^2^/bird) for 28 days	↓	58
		Cross			8 con				
Domestic chicken Single Comb White Leghorn (*Gallus gallus domesticus*)	Adult females (62 weeks)	Expt	Lymphocyte	FISH	12 trt	High stocking density (0.04 m^2^/bird) and restricted food for 14 days	Low stocking density (0.1 m^2^/bird) and ad lib. food for 14 days	↓	105
		Cross/long: 14 days			12 con				
European starling (*Sturnus vulgaris*)	Chicks (3 days)	Expt	Erythrocyte	QPCR	11 trt	Cross‐fostered into broods of seven chicks	Cross‐fostered into broods of two chicks	↓	59
		Long: 11 days			12 con				
European starling (*Sturnus vulgaris*)	Chicks (2 days)	Expt	Erythrocyte	QPCR	17 trt	Cross‐fostered to be 4.8 g lighter than competitor chicks in brood	Cross‐fostered to be 4.9 g heavier than competitor chicks	↓	60
		Long: 9 days			18 con				
Great reed warbler (*Acrocephalus arundinaceus*)	Juvenile birds	Expt	Erythrocyte	QPCR	12 trt	Acute and chronic infection with *Plasmodium ashfordi*	No infection	↓	106
		Long: 9–10 weeks			4 con				
Zebra finch (*Taeniopygia guttata*)	Chicks (1–3 days)	Expt	Erythrocyte	QPCR	30 trt	Brood sizes enlarged by two chicks	No change in brood size	↓	57
		Long: 20 days			25 con				
Coho salmon (*Oncorynchus kisutch*)	Adults (conception)	Expt	Pelic fin (blood and tissue)	QPCR	23 trt	Transgenic fish with enhanced growth rate	Wild‐type	↓	99
		Long: 10 months			15 con				

Expt, experimental; Obs, observational/correlational; long, longitudinal; cross, cross‐sectional; QPCR, quantitative PCR; FISH, fluorescence in situ hybridisation; trt, treatment; con, control; ↓ indicates shorter telomeres (cross‐sectional studies) or greater attrition (longitudinal studies) in stress manipulation group relative to control group.

a For longitudinal studies, age is the age of the subjects at the start of the stress manipulation.

b Durations given for longitudinal studies refer to the period between the first and second telomere measurements.

In contrast to the human literature reviewed above, most of the studies listed in Table 3 involve experimental manipulation of stress, and therefore, for the first time, establish a causal link between organismal stress and TA in vivo. There is some evidence that, as in humans, the effects of stress may be dose‐dependent. Two of the studies contained three ordered levels of their experimental stress manipulation 57, 58, and the graphs presented in these papers suggest graded effects of these levels. Furthermore, although Nettle and coworkers 59 created only two brood size groups, the TA effects reported in their study are actually better predicted by the continuous position of a chick within the weight hierarchy of the brood.

It is striking that some of the stress manipulations in Table 3 produced effects on TL in as little as 9 days 60. These data might be taken to suggest that TA could be used to measure the impact of relatively short‐term stressful events. However, the studies that used the shortest stress manipulations implemented these in very young animals during the period of maximum growth. Such manipulations are likely to produce the largest effects, because age‐dependent TA is greatest during this period, hence magnifying any additional effects of stress. Effects of stress on TA in adults are likely to be smaller than those found in developing animals.

Relatively small numbers of subjects (<30 animals/group) were required to obtain the significant effects on TA reported in Table 3. A contributory factor is likely to be the use of longitudinal measurements, in some cases coupled with sibling designs, which together provide strong controls for individual differences in TL or TA arising from genetics or early development 55, 59, 60, 61, 99. It is also notable that the majority of the studies in Table 3 are on birds. It is possible that the use of erythrocyte TL as opposed to leukocyte TL leads to cleaner estimates of stress‐induced attrition due to the more homogeneous cell population involved.

In summary, there is encouraging preliminary evidence that various types of organismal stress cause quantifiable TA in a variety of vertebrate species.

## A calculus of cumulative experience

**Figure 2:**
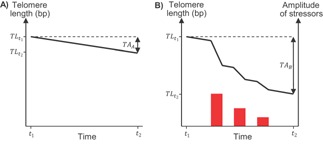
Examples of proposed effects of age and stress on TL between two time points, *t*_1_ and *t*_2_. TA for the time period between *t*_1_ and *t*_2_ corresponds to the difference in TL between these two time points (indicated by the two‐ended arrows). Part **A** shows the change in telomere length over time for an animal that is not exposed to any stressful experiences (i.e. the age‐dependent component of my model). Part **B** shows the change in telomere length over time for the same animal exposed to three stressors (indicated by red bars) each of the same duration but of progressively decreasing amplitude (indicated by the height of the bars). This situation could pertain to an animal that is repeatedly subjected to the same stressful procedure, but that habituates to this procedure such that the amplitude of its stress response decreases with each exposure. Note that the rate of change in TL is greater during the periods of stress and is proportional to the amplitude of the stressors, but returns to the baseline rate of A at the end of each stressor. TA_B_ is greater than TA_A,_ reflecting the greater cumulative severity in B.

The Pickard report on the assessment of cumulative severity in non‐human primates used in neuroscience research concludes: ‘there is no mathematical way of integrating all positive and negative events in an animal's life’ 3. My proposal in this essay is that telomeres provide such a calculus. In Box 1, I present a simple mathematical model based on recent multiple regression analyses of human longitudinal TL data 49. The advantage of a mathematical model is that it forces us to be explicit about the assumptions that we are making – a critical step in devising good tests of the proposal. Specifically, I propose that the number of telomere bp lost over a given time period can be decomposed into two additive components. The first component – age‐dependent TA – is the bp loss attributable to normal cellular turnover during the time period (Fig. 2A; note that this first component could be zero in a species such as the mouse that has adult telomerase expression, or even potentially positive if telomerase expression is especially high). The second component – stress‐induced TA – is additional bp loss attributable to the number, duration and amplitude of stressful events experienced by that individual during the time period. To account for individual differences in stress resiliency, which might arise either due to developmental induction of a more or less resilient phenotype or to the quality of the current environment, I assume that the magnitude of the second, stress‐induced component can be reduced, potentially to zero. Note that the stress‐induced TA due to a specific stressor does not have to be fixed within an individual, but could be altered by learning. This is to allow for the fact that animals might either habituate to specific stressors that are repeatedly experienced, hence losing fewer bp with each successive exposure, or become sensitised, hence losing more bp with each successive exposure (Fig. 2B).

Box 1 **A simple mathematical model describing how age and stress affect TL**

This model is based on recent multiple regression analyses of human data that decompose telomere loss into separate additive effects of age and the numbers of different kinds of stressors experienced, but that additionally find an interaction between life‐style factors and the magnitude of the effect of stressors 49. First, I assume that TL declines with chronological age at a constant rate. This is a simplification, because the rate of age‐dependent telomere attrition is faster in early life in most tissues, but for adult animals and/or for longitudinal studies over a relatively short time period, it is probably a reasonable assumption. Second, I assume that negative experiences can increase TA, and that the additional attrition is proportional to the degree of stress induced by the negative event (its amplitude) and its duration. This assumption follows from the evidence that I have reviewed showing dose‐dependent effects of stressors on TA (see Sections 5 and 7). Third, I assume that the additional attrition caused by negative events can be mitigated to a greater or lesser extent. This assumption follows from the human evidence that the same stressor is capable of inducing more telomere attrition in some individuals than in others 49. Individual differences in resiliency to stress might be explained by genetic differences, differences in the developmental environment and/or differences in the current environment (i.e. life‐style in humans) 49, 107.

We can write down an equation that expresses TL in bp at a follow‐up measurement as TL in bp at a baseline measurement minus attrition due to age‐dependent telomere attrition, minus any unmitigated stress‐induced telomere attrition experienced between the two measurements

$${TL}_{t_{2}}={TL}_{t_{1}}-r(t_{2}-t_{1})-m\sum_{i=1}^{n}\;a_{i}d_{i}$$

where *t*_1_ is the age in years at baseline measurement; *t*_2_ is the age in years at follow up measurement; $TL_{t_{2}}$ is the TL in bp at *t*_2_ (i.e. follow‐up TL); $TL_{t_{1}}$ is the TL in bp at *t*_1_ (i.e. baseline TL); *r* is the age‐dependent TA in bp/year. Note that this term will be positive (implying telomere loss) in species in which telomerase is repressed, but could be equal to zero (or even negative implying telomere gain) in a species such as the mouse in which telomerase is expressed in adult somatic tissues. *n* is the number of discrete stressors experienced between *t*_1_ and *t*_2_. *m* is a multiplier determining the strength of any stress‐mitigating factors due to developmental induction or current environment, for which 0 corresponds to maximum mitigation (i.e. stress‐induced TA is zero) and 1 to the minimum. *a*_1_ is the amplitude of the *i*th stressor experienced expressed as additional TA (bp/year); *d*_1_ is the duration in years of the *i*th stressor experienced.

By comparing age‐dependent TA (TA_A_ in Fig. 2A) with TA measured over the same time period, but with the addition of some form of stressor (TA_B_ in Fig. 2B), it is possible to estimate the additional TA caused by a specific stressor or combination of stressors. We therefore have an objective method for measuring the cumulative severity of any husbandry regime or scientific procedure.

## Conclusions and outlook

I have reviewed evidence that TA meets the criteria for a good measure of cumulative experience that could be used to assess the welfare impact of husbandry regimes and/or experimental procedures on non‐human animals. My review establishes that TL is objectively measureable via minimally invasively acquired samples in any species of welfare concern; TL is sensitive to the cumulative effects of negative experience in a dose‐dependent manner, more negative experiences being associated with greater telomere loss; TL may also be sensitive to positive experiences, positive experiences being associated with mitigation of the effects of negative experience; finally, TL is positively correlated with more positive mood in humans, validating its use for measuring the valence of experience in non‐human animals.

I propose that the cumulative severity of a procedure or husbandry regime could be expressed in terms of the additional cellular ageing it imposes (measured in bp or years). Increased biological age provides a readily interpretable common currency in which to compare the relative impact that different kinds of events have on an animal. There exists encouraging preliminary evidence from non‐human animals supporting this proposal. However, further studies in animals are required: to replicate these findings and establish their generality across species (especially mammals and fish); to explore whether exposure to short‐term acute stress produces quantifiable effects on TA in adult animals as well as young animals; to establish whether there are dose–response relationships between number and intensity of stressors and TA; and to test experimentally whether TA can be mitigated or reversed via positive welfare interventions, such as provision of environmental enrichment. Future experimental work should adopt powerful experimental designs, involving both longitudinal measurements and ideally additional controls for genetic variability, to maximise the chances of detecting effects of organismal stressors on TA.

The author has declared no conflicts of interest.
